# Case of Communication between the Ventricles of the Heart;—the Aorta Originating from Both Ventricles

**Published:** 1845-05

**Authors:** 


### MEDICAL EXAMINER,

AND

### RECORD OF MEDICAL SCIENCE.

NEW SERIES—No. V.—MAY, 1845.

### ORIGINAL COMMUNICATIONS.

*Case of communication between the Ventricles of the Heart;—the Aorta originating from both Ventricles.* Communicated by Professor Dunglison.

[The following case is not unique. Yet the malformations are uncommon, and we are not aware that the subjects have lived so long. Generally, they have died before or about the time of puberty. Rarely have they survived the changes that take place at that epoch; and commonly, as in this case, disease has supervened in some other important organ, the predisposition being caused by irregularity of circulation, owing to the malformation in the great central organ. Throughout life, black blood must have been sent into the aorta; yet, under ordinary circumstances, no cyanosis was apparent. When, however, irregularity was induced in the circulation by any cause, the blueness was perceptible. As the ventricles contract together, but little admixture of the blood of the two sides of the heart appears to have taken place through the inter-ventricular communication. The case confirms the belief, now almost universal, that imperfectly oxygenized and decarbonized blood is not of necessity seriously morbific; and where the pulmonary artery is unusually small, the admixture of venous and arterial blood, owing to the patulous state of the foramen ovale, or other cause, admitting of such admixture, may even be the means of prolonging life.]

*Worcester*, April 2d, 1845.

To Professor R. Dunglison.

Dear Sir,—In compliance with your request, I send you the particulars, so far as I have been able to collect them, of the case of malformation of the heart, of which I spoke when I saw you last. The girl was Irish, 21 years of age, and had been in the Worcester alms-house about a year, previous to which I know nothing of her history. During her residence there, she was unable to perform any work, or take exercise of any kind, as upon the least exertion or excitement she was attacked with palpitation and dyspnœa, accompanied with some blueness of the skin, all which gradually disappeared on her remaining at perfect rest. She was also subject to attacks of the same kind after dinner, which, during the latter part of her life, came on every day, continuing for two or three hours, even though she remained perfectly quiet, being apparently excited by the mere stimulus of food. She had also a constant and strong rasping *bruit*, accompanying the first sound of the heart, and masking it partially. She suffered much from headache, which, a few days before her death, became greatly increased, accompanied with high febrile excitement, followed by delirium, coma and death. On examination, the septum ventriculorum was found deficient just at the orifice of the aorta, giving that vessel an origin from both ventricles; the pulmonary artery was contracted so as barely to admit the little finger; the lungs were of natural colour, but both were studded from top to bottom with crude and miliary tubercles, none of which were softened; the left lateral ventricle of the brain was filled with pus, the lining membrane being inflamed and thickened, and in the posterior lobe of the same hemisphere was found a cyst the size of a robin's egg, also filled with pus, but having no connection with the ventricle. The substance of the brain presented numerous red points, but was not altered in consistence. No other morbid appearances whatever were observed in the organs examined, which were the lungs, heart and brain. At the time of her death she was menstruating profusely. Blueness of surface was at no time considerable; and it was only observed upon some exertion or excitement, and always in connection with palpitation and dyspnœa,—coming on and disappearing with them.

I regret that I cannot furnish you more minute notes of the case; but should I have omitted anything you may desire to know, I shall be most happy, on the receipt of a line from you to that effect, to give you all the additional information in my power. I should be exceedingly glad to receive your views of the case, and any reflections it may suggest.

With much respect, I am sincerely yours,

Charles Bertody.

